# The OxyCam: A novel hand-held vital microscope for quantitative measurement of microcirculatory perfusion and oxygenation

**DOI:** 10.1016/j.jointm.2026.01.010

**Published:** 2026-04-03

**Authors:** Can Ince, Bülent Ergin, Jonathan Montomoli, Mario Döring, Noor Boegborn, Wijnie van Dam, Giovanny Marchena, Olcay Dilken, Arie de Vos, Husain Khaki, Diederik Gommers, Matthias Peter Hilty

**Affiliations:** 1 The Department of Intensive Care, Laboratory of Emeregency, Perioperative and Intensive Care (EPIC lab), Erasmus MC, University Medical Center, Rotterdam, The Netherlands; 2 Department of Anaesthesia and Intensive Care, Infermi Hospital, Romagna Local Health Authority, Rimini, Italy; 3 Active Medical B.V., Leiden, The Netherlands; 4 University Hospital of Zurich, Institute of Intensive Care Medicine, Zurich, Switzerland

**Keywords:** OxyCam, Cytocam, Sublingual, Spectrophotometry, Tissue oxygenation, Tissue perfusion

## Abstract

**Background:**

Hand-Held Vital Microscopy (HVM) has been utilized in clinical investigations to measure microcirculation, providing valuable insights into the pathophysiology of critical illness and its treatment. However, its clinical implementation has been hampered by several shortcomings. These included the need for automatic analysis software, for instant image stabilization, and for information regarding microcirculatory oxygenation. The aim of this study is to describe an HVM which has realized these research objectives.

**Methods:**

This preclinical study introduces the OxyCam, a novel HVM device. OxyCam stabilizes images during recording and subsequently provides essential microcirculatory functional parameters through automatic analysis. Green and blue illuminated images, sequentially recorded and ratio imaged, allow calculation of the hemoglobin (Hb) oxygen saturation (µHbO_2_sat) of RBCs and are presented in pseudo color images. We validated the OxyCam on the hindlimb muscle of 9 male Wistar albino rats, mechanically ventilated at different levels of inspired oxygen (FiO_2_). Comparison was made between OxyCam-green and blue images and to its predecessor the Cytocam. OxyCam-generated µHbO_2_sat values were compared to HbO_2_sat fiber spectrophotometry (O2C).

**Results:**

Green and blue illumination from the OxyCam yielded higher total vessel density (OxyCam green [31.4±4.3] mm/mm^2^; OxyCam blue [32.5±4.2] mm/mm^2^, Cytocam [28.5±3.6] mm/mm^2^) and functional capillary density (OxyCam green [25.4±6.4] mm/mm^2^; OxyCam blue [26.2±7.9] mm/mm^2^; Cytocam [23.0±4.5] mm/mm^2^) than the Cytocam images OxyCam-blue gave higher tissue RBC perfusion values (OxyCam green [55.3±18.0] µm/min; OxyCam blue [69.5±25.5] µm/min; Cytocam [58.3±15.3] µm/min) than the OxyCam-green or Cytocam images. OxyCam-blue and green images gave better image quality than the Cytocam images. µHbO_2_sat imaging with the OxyCam was validated by demonstrating expected changes in µHbsat during different FiO_2_ levels (100%, 60%, 21%, 0%). Furthermore, a correlation between the mean µHbO_2_sat of images and independently measured µHbsat by tissue spectrophotometry during different levels of FiO_2_ was found. Finally, µHbO_2_sat images in pseudo-color (red to blue) are shown for the individual microvessels at different FiO_2_ levels.

**Conclusions:**

The OxyCam has been validated for measuring tissue perfusion and oxygenation and can be considered for point-of-care diagnosis of the microcirculation at the bedside.

## Introduction

Shock is defined as circulatory failure, resulting in the inability of the circulation to provide adequate tissue perfusion and oxygenation to meet the metabolic needs of tissue cells for their survival and to support organ function. This definition, dates back to 1920^[^^1^^]^ and persists to date in the current consensus on shock definition.^[^^2^^,^^3^^]^ Indeed, many resuscitation procedures, such as fluid therapy, blood transfusion and vasoactive therapy define the restoration of tissue perfusion as the ultimate goal of therapy.^[^[4], [5], [6]^]^

Despite general agreement on the importance of tissue and microcirculatory perfusion and oxygenation in critical care medicine, no validated clinical bedside technique exists to quantitatively and automatically characterize microcirculatory tissue perfusion and oxygenation at the bedside. Instead, surrogate markers, such as lactate, arterial-venous carbon dioxide difference, peripheral temperature, near infrared spectroscopy (NIRS), and capillary refill time, are used.^[^^7^^]^

Alterations in sublingual microcirculation using various generations of hand-held vital microscopes (HVM) has been used over the past decades for direct observation of red blood cells (RBC) perfusion of the microcirculation in disease and therapy.^[^^8^^]^ HVM observations provide quantitative information about RBC perfusion and allows a distinction to be made between the diffusive and convective properties of microcirculatory perfusion.^[^^9^^,^^10^^]^ Although sublingual HVM has been widely used to gain insight into the identification of a loss of hemodynamic coherence between the macro and microcirculation,^[^^11^^]^ the nature of sepsis shock and resuscitation, and as a sensitive indicator of outcome,^[^[12], [13], [14], [15]^]^ it has not gained wide acceptance as a routine point of care bedside clinical tool.^[^^16^^]^

The 2018 International Consensus on the assessment of sublingual microcirculation identified the requirements for the future development of HVM to become a routine bedside diagnostic tool.^[^^10^^]^ The first main requirement was the need for a clinically validated point-of-care automatic analysis platform.^[^^10^^]^ We recently developed such an automatic software platform called MicroTools and clinically validated it in a large international database of patients.^[^^9^^]^ The second main requirement of the 2018 Consensus was the need to measure the amount of oxygen being transported by RBC in the microcirculation.^[^^10^^]^ This is important since physiology dictates that RBC-encapsulated hemoglobin (Hb) is the primary passage by which oxygen is transported to the tissues to meet their metabolic requirements.^[^^17^^]^

To meet the above requirements, we developed a novel generation HVM called the OxyCam. To measure the oxygen saturation in the RBCs in the microcirculation we applied the dual wavelength illumination of Hashimoto to image the distribution of microcirculatory Hb oxygen saturation (µHbO_2_sat) in the vessels.^[^^18^^]^ For the purposes of the OxyCam, we developed an altered version of MicroTools which we have called OxyTools to accommodate dual wavelength oximetry and introduced an instant stabilization algorithm, which allowed rapid access to the image for automatic analysis,. In doing so, the functional properties generated by the embedded OxyTools of the OxyCam fulfil the requirements of the 2018 Consensus on Sublingual Microcirculation for a point-of-care clinical HVM device for bedside use.^[^^10^^]^ Its validation is described in this paper.

## Methods

### Description of the OxyCam

OxyCam is designed as most HVM systems are, with a computer control image sensor attached to an image guide consisting of a set of lenses covered by a disposable plastic medically clean cap. The optical system of the lens pipe consists of a set of specially designed high-resolution lenses considering the optical properties of the different wavelengths needed for dual wavelength oximetry in a lens pipe with a magnification factor of x4.4. The tip of the lens pipe is placed on an organ surface, and images of the microcirculation under the surface are projected onto a high-resolution image sensor. The high-resolution digital image sensor consists of 2472 × 2064 pixels, with a pixel size of 2.74 µm^2^ and is used to record image sequences. To avoid surface reflections, illuminating 8 light emitting diodes (LED) are placed around the circumference of the tip of the lens pipe of which four are green (527 nm) and four are blue (470 nm). In this way, Incident Dark Field (IDF) illumination is accomplished according to Sherman and Cook^[^^19^^]^ as well as direct illumination. The tissue surface can be illuminated with either green or blue light or in oximetry mode, alternating sequentially between the two wavelengths. The OxyCam is attached to a clinically certified Linux-based tablet fitted with a graphical user interface (GUI) for control of image acquisition, focus, brightness, the frequency, and duration of the blue and green light illumination, or alternatively to a MacBook Pro computer. Acquired images are then processed by OxyTools for automatic quantitative calculation of all the functional microcirculatory parameters, as well as the tissue red blood cell perfusion (tRBCp)^[^^20^^]^ and microcirculatory (µHbO_2_sat) seen in the FoV. Calculation of the µHbO_2_sat in each RBC position in the vessels are conducted by ratio imaging of the superimposed two wavelength images and presented as pseudo-colors superimposed on the microcirculation images of the flowing RBC in the FoV.

Focusing is accomplished by movement of the image sensor by a software-controlled stepper motor with a step size of 20 µm. This feature allows quantitative depth of focus measurements of the microcirculation with respect to the tissue surface to be made.^[^^21^^]^ We showed that such a measurement can be used for quantitative measurement of fluid resuscitation-induced tissue edema in burn patients.^[^^22^^]^ This feature also allows easy sequential measurements to be made without the need to refocus.

### The OxyTools GUI

**Figure 1:**
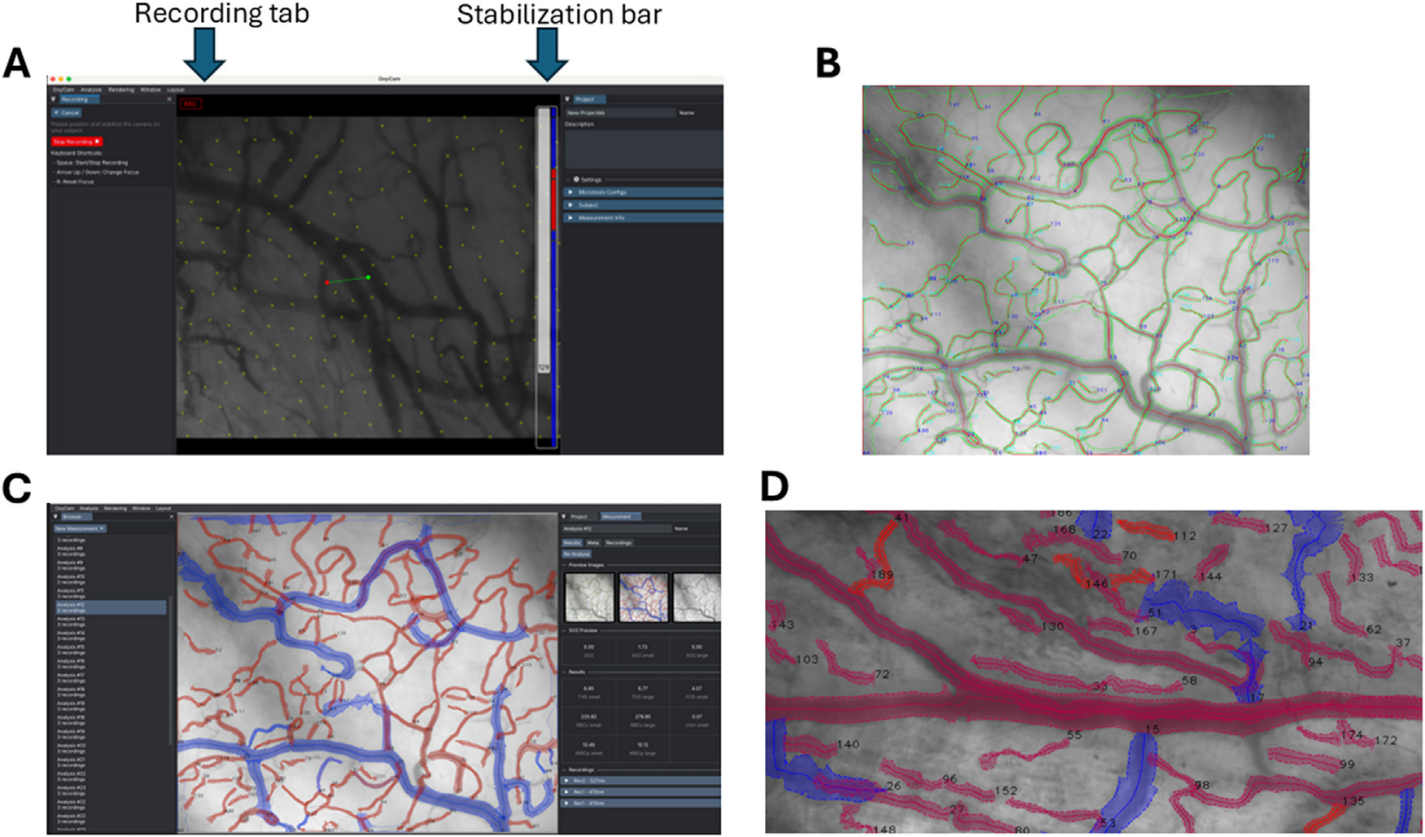
Examples of the graphic user interface. A: The acquisition is started by pressing the record button, and the automatic stabilization is initiated, which can be followed by the movement of the cursor on the vertical stabilization (shown by the arrows). Stabilization is completed when the stabilization of the cursor has reached the bottom of the stabilization bar. B: The analysis phase is initiated by segmentation of the vessels (software recognition of the contours of the vessels) C: followed by calculation of the microcirculatory and oxygenation parameters acquisition. In this phase, the recorded image sequence can be observed in blue or green illumination, or the calculated hemoglobin satturation image drawn in pseudocolors (red well saturated, blue poorly saturated red blood cells). D: shows an amplified section of the microcirculatory µHbO_2_sat images of a field of view. Note that each vessel is identified by a number which can be identified. In the circled areas, a red capillary (vessel 189) can be seen flowing into an intermediate venule (vessel 41), which flows downstream in the terminal venule (vessel 17), which is colored blue. In the data sheet associated with this image, µHbO_2_sat value is given of the beginning of a vessel and the value at its distal, from which the tissue oxygen extraction can be calculated for each individual vessel. µHbO_2_sat: Hemoglobin oxygen saturation.

The purpose of the GUI is to control the recording and analysis functions of the OxyCam and display them on the computer screen for interactive communication with the operator. The GUI comprises five phases: (1) the focusing phase, (2) the stabilization and recording phase, (3) segmentation of the vessels, (4) analysis phase providing microcirculatory perfusion and Hb saturation parameters, and (5) the export function of the images and data. Three screenshots of 2–4 associated with the GUI are shown in Figure 1.

The recording phase of the GUI follows placement of the tip of the OxyCam on the tissue surface to obtain a well-focused image of the microcirculation, making sure a mix of capillary and venule vessels are observed in the FoV when used sublingually.^[^^23^^]^ Focus is accomplished by movement of the stepper motor till sharp images of moving RBC in the capillaries in the FoV are seen. A default recording time of 4 s is made although longer recording times is possible by change of the configuration settings. The GUI is fitted with an instant stabilization module which stabilizes the image during recording in the acquisition phase of the image (Figure 1A). This instant stabilization option is an important advance on previous HVM devices, which required stabilization of the image offline and, if not successful, required repeated measurements on the patient until a suitable stable image was made. These features, whereby OxyCam acquires a stabilized image and allows instant analysis, substantially reduces the measurement time and analysis time needed for obtaining point-of-care information regarding the functional state of the microcirculation in the FoV from some 30 min using conventional HVM to several seconds in the present GUI.

The OxyTools software incorporated in the OxyCam consists of the MicroTools clinically validated automatic software analysis, for instant analysis of microcirculatory image sequences for providing quantitative parameters related to the functional activity of the microcirculation in the FoV, plus the dual wavelength oximetry software to calculate and present graphically µHbO_2_sat colors superimposed on the micro vessels. Segmentation of the acquired images for identification of the blood vessels is achieved by MicroTools routines.^[^^24^^]^ The quantitative functional parameters of the microcirculation related to the diffusive component of the microcirculation include total vessel density (TVD) and the functional capillary density (FCD).^[^^9^^,^^10^^]^ In addition to these functional parameters, an important new functional parameter combining the diffusive and convective components of microcirculation called tRBCp is included.^[^^20^^]^ The tRBCp is a quantitative parameter (µm/min) which describes the oxygen delivery capacity of the RBC in the microcirculation to the tissues.

Analysis of the green and blue images allows calculation of the µHbO_2_sat. OxyTools measures the luminosity of each pixel and calculates the optical density within and outside the length of each micro vessel from which the µHbO_2_sat is calculated.^[^^18^^]^ OxyTools and the GUI present the microcirculatory moving images of the flowing red and white blood cells^[^^25^^]^ together with a compiled microcirculatory image superimposed on the micro vessels in pseudo colors ranging from red (well saturated µHbO_2_sat) to blue saturated (de saturated µHbO_2_sat). Leucocytes do not effect to the calculation of µHbO_2_sat because they do not contain Hb.

### Validation of microcirculatory (µHbO_2_sat) imaging

Validation of the change in microcirculatory Hb saturation was accomplished by comparison the mean µHbO_2_sat measured by fibre spectrophotometry (O2C, Lea Medizintechnik GmbH, Germany). The O2C device is a spectrophotometry device that is connected to a fibre whose tip is placed on tissue surfaces. The fibre consists of a light guide with concentric illumination of the tissue surface with white light. The O2C device analyses the reflected light by a spectrophotometer, producing a spectrum of the reflected light. This reflected spectrum, from 400 nm light to 600 nm, is then computer-analysed to calculate the microcirculatory Hb saturation of the microcirculation under view. We have previously validated the O2C device in hemodiluted pigs by comparing the O2C microcirculatory µHbO_2_sat signal to quantitative microcirculatory oxygen pressure measurements using palladium porphyrin quenching of phosphorescence^[^^26^^,^^27^^]^ and have applied the technique in clinical sublingual oximetry studies.^[^^5^^,^^28^^]^ In the present study, the mean µHbO_2_sat value measured by the O2C device was used as a gold standard for comparison to the mean µHbO_2_sat of the images obtained from the OxyCam. The catchment area of the O2C fibre is similar to that of the FoV of the HVM device. Different values of inspired oxygen (FiO_2_) were then applied to the ventilator to generate differing values of µHbO_2_sat of the hind limb muscle. OxyCam measurements were alternated with O2C measurements.

### Rat hindlimb muscle validation experiments

This study was approved by the National Committee of Animal Experimentation (PPL2317333) and the Animal Welfare Body of Erasmus Medical Center, Rotterdam (SP2300253). Nine male Wistar albino rats with bodyweight of (416 ± 21) g were used (Charles River, The Netherlands). The number of animals used for the study was determined by the power analysis of differences between two dependent means (matched pair) (G Power, HHU, Germany) with a TVD mean 1 of 30 mm/mm^2^ and a standard deviation of 4, and an estimated mean 2 of 33 mm/mm^2^, a standard deviation of 3. The effect size, alpha error, and power were calculated as 0.554, 0.050, and 0.500, respectively. A total of 36 pair comparisons were needed, corresponding to 9 animals with 4 time points. Care and handling were performed in accordance with the institutional and Animal Research: Reporting of In Vivo Experiments (ARRIVE) guidelines.

Rats were instrumented, anesthetized and mechanically ventilated as described previously.^[^^29^^]^ The biceps femoris muscle was exposed, and the tip of the lens pipe of the Cytocam, as the predicate hand-held vital microscope (Cytocam, Braedius, The Netherlands), followed by OxyCam, was gently placed on the exposed biceps femoris muscle. Care was taken to place the tip of the devices on the same location on the muscle surface. Both devices were optimally focused to achieve good visualization of the flowing single red and white blood cells in the capillaries of the muscle microcirculation. Using the Cytocam and the OxyCam in this manner, several time points were measured whereby each time point consisted of three image sequences, a Cytocam (green illumination) measurement and an OxyCam green and blue illumination measurement. Each experiment was a terminal experiment for the animal and lasted approximately 6 h. A total of 9 rats were examined with four different FiO_2_ levels (100%, 60%, 21%, and 0%). Eight of those were included for the evaluation of microcirculatory parameters of green and blue illuminated OxyCam and green illuminated Cytocam images (giving a total of 32 images, which were analyzed). Four rats were used for analysis of μHbO_2_sat measurements using the OxyCam and O2C at 4 different FiO_2_ levels (100%, 60%, 21% and 0%) achieved by cessation of ventilation (giving a total of 16 measurements) on the same spot of the biceps femoris muscle. The experiment was terminated by euthanization by intravenous 1 mL/kg Euthasol (20% pentobarbital) infusion.

### Protocol

After 30 min of stabilization of the rats, the Cytocam tip of the light guide was gently placed on the muscle surface, and a 4-s image clip was recorded at 100 frames per second during each FiO_2_ level. Then the tip of the OxyCam was placed on the muscle, and frames of green light illumination were recorded, and next a similar clip of blue light illumination was recorded. Images were analyzed by OxyTools, providing the microcirculatory parameters listed above. Oximetry values calculated include µHbO_2_sat expressed as oxygen saturation in each of the individual vessels (presented in pseudo-color and available as% HbO_2_sat) as well as the mean µHbO_2_sat values of the whole field of view.

### Quality of images

To compare the quality of microcirculatory images obtained by the green illumination of Cytocam and the green and blue illumination of the OxyCam, a Python script was written using OpenCV and NumPy to compute the Laplacian variance for sharpness, the standard deviation for contrast and the mean for brightness of the whole image seen in the field of view as described elsewhere.^[^^30^^]^ The vessel quality of the entire image was defined as the multiplication of the normalized contrast and the normalized sharpness, as was described previously by Goedhart et al.^[^^31^^]^

### Statistics analysis

We analyzed microcirculatory values repeatedly in the same animal under three modalities (OxyCam Green, OxyCam Blue, Cytocam). For analysis, the primary model used was a linear mixed-effects model (LMM) with a fixed effect for HVM and a random intercept for subject to account for within-subject correlation.

Models were fit by restricted maximum likelihood; denominator degrees of freedom were obtained via Satterthwaite approximation. Model assumptions were assessed by inspection of residual diagnostics. We estimated marginal means for each measurement. All pairwise differences among HVM levels were reported with Tukey adjustment. A two-sided α=0.05 was used for hypothesis tests. All analyses were performed with R Software (version 4.4.1. R Foundation for Statistical Computing, Vienna, Austria).

## Results

### GUI

**Figure 2:**
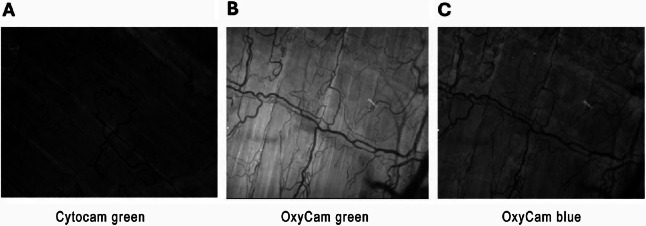
Examples of the rat hindlimb muscle microcirculation taken with the Cytocam (A: green illumination), OxyCam (B: green illumination), and OxyCam (C: blue illumination).

The GUI, which controls the OxyCam, is visualized on the screen and can be controlled by the operator. The tip of the OxyCam is placed on a tissue surface where a suitable mix of capillaries and venules are observed. Care is taken not to exert excessive pressure on the tissue surface as indicated by obstructed venular RBC flow as stated by the European Society of Intensive Care Medicine (ESICM) guidelines on sublingual microcirculatory.^[^^10^^]^ Prior to the image being recorded, focus is achieved by GUI control of the stepper motor till single flowing RBC can be seen sharply in the capillaries. Then, the recording is initiated by pressing the record button, and the automatic stabilization is initiated, which can be followed by the movement of the cursor on the vertical stabilization bar. Stabilization is completed within seconds if the operator is suitably trained and is indicated by the cursor reaching the bottom of the stabilization bar (Figure 1A). This concludes the acquisition phase, and the recorded clip is ready for analysis. The recording phase includes the segmentation of the vessels (software recognition of the contours of the vessels) (Figure 1B) and then calculation of the microcirculatory and oxygenation parameters of the observed microcirculation in the FoV. In this phase the recorded image sequence can be played back for inspection in blue or green illuminated images, or the calculated µHbO_2_sat image can be observed with drawn in pseudo colors (red well saturated blue poorly saturated RBC) in the various micro vessels (Figure 1C). As can be seen in a detail of a µHbO_2_sat image (Figure 1D) each vessel is numbered, and upstream capillaries are redder than the larger distal venules as would be expected as oxygen leaves the vasculature into the tissues. Figure 1D also shows how a redder capillary can be seen flowing into an intermediate venule flowing downstream in the terminal venule which is colored blue. Each vessel is identified by a number which can be identified (Figure 1D) in a data sheet where all the functional parameters related to the perfusion and oxygenation of each individual vessel can be found and outputted as a data file. Regarding the µHbO_2_sat measurements, the value can be obtained at the beginning of a vessel and at its distal end from which the tissue oxygen extraction can be calculated per vessel Figure 2.

### Image quality

The quality of the three different modalities were compared to each other for resolution, sharpness, and quality of image (Table 1). The results showed that OxyCam outperformed Cytocam in terms of the quality of image, both in green and blue.

**Table 1:** Quality of image score based on the measurement of sharpness, contrast, and brightness. The OxyCam, both in green and blue, provides a better quality of image than the Cytocam.

Modality	Sharpness	Contrast	Brightness	Quality
OxyCam green	0.29 ± 0.08	0.12 ± 0.03	0.72 ± 0.07	0.040 ± 0.020
Cytocam	0.03 ± 0.01	0.08 ± 0.03	0.36 ± 0.12	0.003 ± 0.000
OxyCam blue	0.13 ± 0.04	0.09 ± 0.03	0.53 ± 0.07	0.010 ± 0.000

### Comparison of microcirculatory parameters

**Figure 3:**
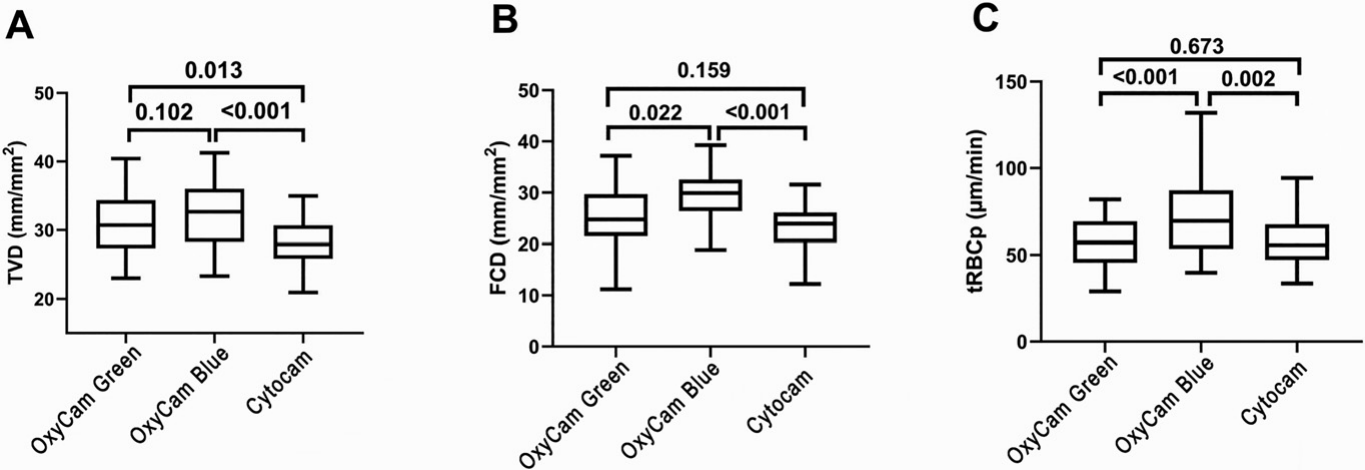
OxyTools-based automatic analysis and comparison of microcirculatory images acquired by OxyCam under green illumination, OxyCam under blue illumination, and the reference Cytocam device across eight rats. A: total vessel density (TVD), B: functional capillary density (FCD), C: tissue red blood cell perfusion (tRBCp). Numbers indicating between-group comparisons correspond to *P* values. FCD: Functional capillary densit; tRBCp: Tissue red blood cell perfusion; TVD: Total vessel density.

OxyCam can generate both green and blue illuminated images. In this way, a comparison was made between the microcirculatory parameters calculated from the green and blue illuminated OxyCam images and, compared to the microcirculatory parameters calculated from the Cytocam (green illumination) images (Table 2). The TVD of the Cytocam was compared to that of the OxyCam green illuminated images in 8 rats. Results showed that the two systems gave comparative values of TVD (Figure 3A) calculated by the validated automatic MicroTools software embedded in the OxyCam. Results, as shown in Figure 3, indicated that the TVD calculated from OxyCam images illuminated with green and blue light yielded a higher TVD than that calculated from the green-illuminated Cytocam images (*P*=0.013 and *P<*0.001, respectively). The green and blue illuminated OxyCam images provided equivalent values of TVD (Figure 3A) (*P*=0.102).

**Table 2:** Comparison of microcirculatory parameters obtained with OxyCam (green and blue illumination) and Cytocam.

Parameter	OxyCam (Green)	OxyCam (Blue)	Cytocam
TVD (mm/mm²)	31.4 ± 4.3	32.5 ± 4.2	28.5 ± 3.6
FCD (mm/mm²)	25.4 ± 6.4	26.2 ± 7.9	23.0 ± 4.5
Tissue RBC perfusion (µm/min)	55.3 ± 18.0	69.5 ± 25.5	58.3 ± 15.3

Data are presented as mean ± standard deviation.

FCD: Functional capillary density; RBC: Red blood cell; TVD: Total vessel density.

Comparison of the diffusive component of the microcirculation, the FCD, since no difference was found between the OxyCam green and Cytocam green illumination (*P*=0.159), OxyCam blue images gave higher FCD values than both OxyCam green and Cytocam green image values (Figure 3B) (*P*=0.022 and *P<*0.001, respectively). The convective component of the microcirculation, tRBCp, showed no significant differences between the OxyCam green and Cytocam green (*P*=0.673), but it showed a higher value for the OxyCam blue images than either the OxyCam green or the Cytocam green images (*P<*0.001 and *P*=0.002, respectively) (Figure 3C).

### Microcirculatory HbO_2_sat measurements

**Figure 4:**
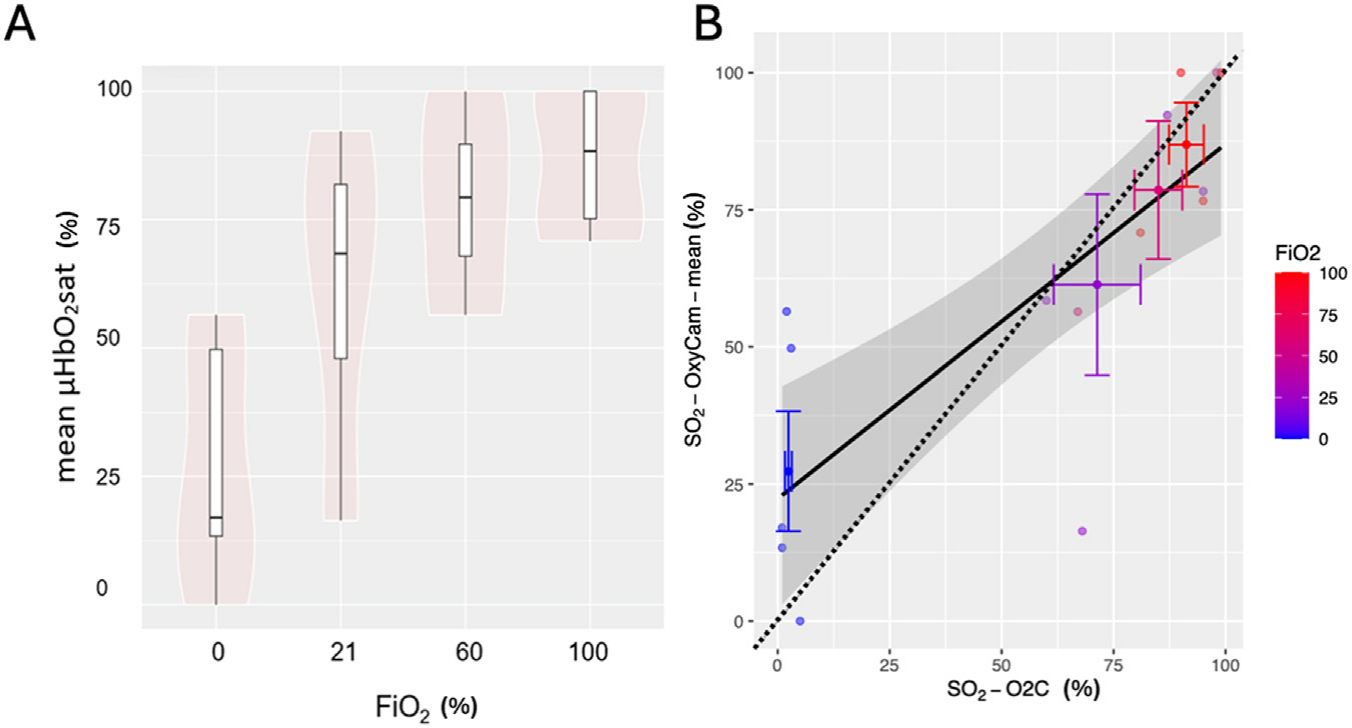
Validation of OxyCam microcirculatory µHbO_2_sat imaging dependency on inspired oxygen fraction (FiO_2_) against controlled oxygenation and a comparative O2C method in a ventilated rat hind limb model (*n*=4). A: Validation of OxyCam response to systemic oxygenation changes. The mean µHbO_2_sat measured by OxyCam imaging increased predictably with elevated inspired oxygen fraction (FiO_2_) in the mechanically ventilated rat hind limb model (*n*=4). B: Correlation between OxyCam imaging and fiber spectrophotometry (O2C). µHbO_2_sat was measured simultaneously on the same muscle using OxyCam and O2C. Individual data points (symbols coded by FiO_2_ level) are plotted alongside the linear regression (dark line; shaded area represents confidence interval). The cross indicates the overall mean ± standard error of the mean. Deviation of the regression line from the identity line suggests that the O2C signal, limited by its lower sensitivity and signal-to-noise ratio in low-flow states, may not fully capture saturation in the smallest vessels detected by OxyCam. The data also reflect the challenge of achieving complete muscle deoxygenation via hypoxic ventilation alone. Despite this, a strong correlation exists between the two techniques.

To demonstrate the ability of the OxyCam to measure µHbO_2_sat, we applied the ratio imaging methodology described by Hashimoto to calculate µHbO_2_sat on the green and blue images of the OxyCam.^[^^18^^]^ The mean value of µHbO_2_sat of the whole FoV was calculated at various levels of FiO_2_. Figure 4A shows the dependency of the mean muscle tissue µHbO_2_sat on inspired FiO_2_ levels in a predictable manner.

To further validate the dual wave ratio imaging methodology embedded in the OxyCam, the calculated mean µHbO_2_sat in images of all the vessels in the FoV using OxyTools at different FiO_2_ levels were compared to those independently measured by O2C spectrophotometry fibre measurements. Results showed a good correlation between the OxyCam-generated ratio imaging-calculated µHbO_2_sat and the O2C-measured µHbO_2_sat (*r*=0.79 and *P*=0.0002) (Figure 4B).

**Figure 5:**
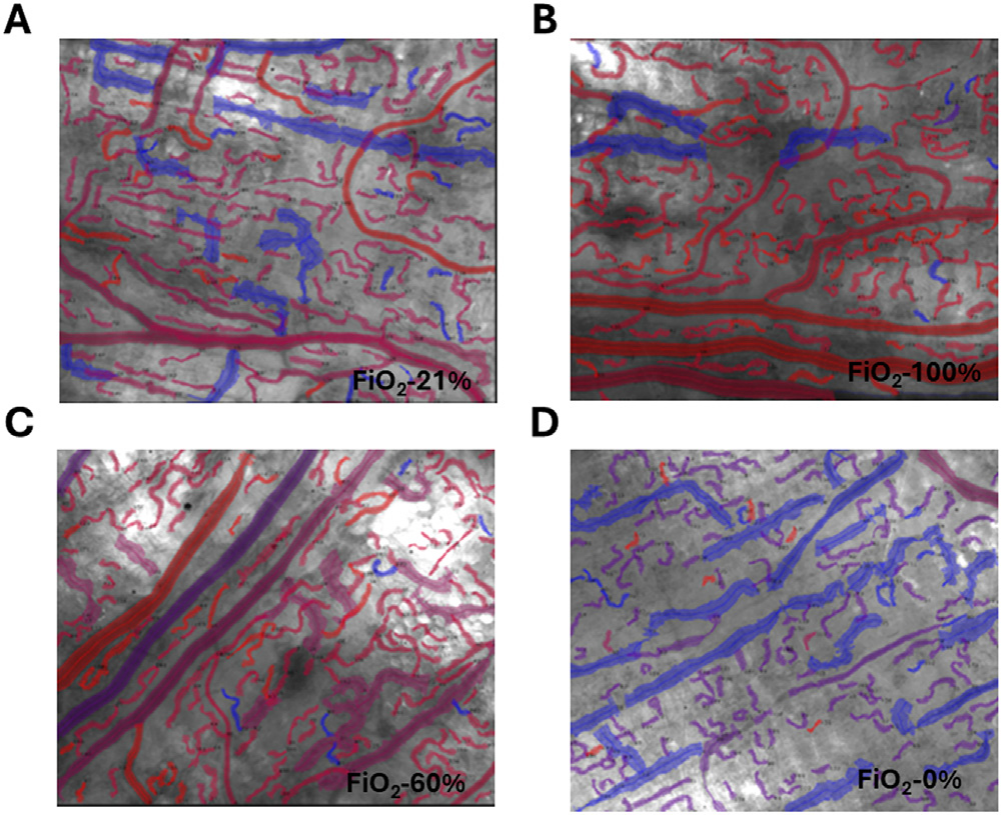
Representative microvascular µHbO₂sat maps obtained by OxyCam two-wavelength ratio imaging under varying FiO₂. A: FiO₂ 21%. The µHbO₂sat map exhibits a mixed pattern, with well-saturated capillaries (red) transitioning to lower saturation in distal venules (blue), consistent with expected oxygen diffusion along the capillary-venule axis. B: FiO₂ 100%. The microvascular network appears predominantly red, indicating uniformly high hemoglobin oxygen saturation throughout the visualized vessels. C: FiO₂ 60%. An intermediate saturation pattern is observed, featuring a greater proportion of red capillaries alongside progressively desaturated venules (blue). D: FiO₂ 0%. The map is dominated by blue, reflecting severe hemoglobin desaturation across the entire microcirculatory network. µHbO₂sat: Hemoglobin oxygen saturation; FiO₂: Inspired oxygen fractions.

Figure 5 shows examples of the images at the various levels of FiO_2_. The drawn in color scheme goes from blue (low saturation) to red (high oxygen saturation). As can be seen in the images in Figure 5A at 21% FiO_2_, a mix of red and blue vessels are seen. The smaller capillary vessels are upstream and, as expected, are redder associated with a higher saturation than the downstream larger venules, which are, also as expected, blue. Increasing the inspired oxygen to 100% results in almost all vessels becoming red and fully oxygen saturated (Figure 5B), whereas reducing the FiO_2_ to 60% results in vessels becoming less saturated, especially the downstream venules (Figure 5C). Note the intermediate purple vessels between the capillaries and the large venule. Secession of the ventilation results in the muscle becoming hypoxic with all the vessels becoming blue (Figure 5D). Together these results show that the ratio imaging methodology using green and blue illumination works. They can measure tissue µHbO_2_sat using the OxyCam reliably both at a morphological level and as mean µHbO_2_sat values taken over the whole FoV.

## Discussion

In the present study, an HVM, called the OxyCam, designed to measure quantitatively microcirculatory perfusion and oxygenation, was validated in the hind limb muscle of mechanically ventilated rats and compared to its predecessor HVM, the Cytocam. Instant image stabilization, together with the embedded OxyTools, considerably shortens the acquisition and analysis time compared its predecessor HVM, which requires offline processing and analysis. Subsequently, the OxyTools is able to automatically analyse the microcirculatory perfusion parameters, as well as the oxygen saturation values of the RBCs flowing in the microvessels, using dual-wavelength ratio imaging oximetry.

Previous generations of HVM devices have been developed, including orthogonal polarization spectral imaging (OPS), sidestream darkfield imaging (SDF), and IDF devices,^[^^32^^]^ and these have been validated in various studies.^[^^31^^,^^33^^,^^34^^]^ The most advanced HVM currently in use is the Cytocam although, due to its lack of validated automatic software and absence of oximetry it has not met the requirements of the 2018 International Consensus on the assessment of sublingual microcirculation for it to be used as a routine clinical point of care diagnostic tool.^[^^10^^]^ Nevertheless the Cytocam has been shown to be able to detect 30% more vessels than in its previous version, SDF imaging.^[^^34^^]^ which is why we chose the Cytocam as a predicate device to compare it to the OxyCam in the present study.

Comparison was made between the image quality, contrast, brightness, and sharpness of the green and blue illuminated OxyCam images. Results showed comparable image quality between OxyCam green illumination and OxyCam blue illumination, whereas the OxyCam outperformed the Cytocam in terms of image quality (Table 1). The functional parameters measured from OxyCam green and blue illuminated images were compared to each other and to those obtained by the green illuminated Cytocam images. Results showed that TVD measured with the OxyCam provided higher values than those of the Cytocam, but FCD and tRBCp were comparable between the OxyCam and Cytocam green illumination. Additionally, blue-illuminated OxyCam images yielded higher TVD, FCD, and tRBCp than green-illuminated Cytocam images. These findings are consistent with the composite findings that the OxyCam lenses provide better image quality (Table 1), in combination with earlier findings that blue illumination provides better image quality than green illumination.^[^^35^^]^

The ratio-imaging method, introduced by Hashimoto for µHbO_2_sat oximetry, was successfully incorporated into the OxyCam. Mean µHbO_2_sat values followed the levels of inspiratory oxygen as expected. Images of µHbO_2_sat produced pseudo color images of the µHbO_2_sat of the different generation micro vessels in a similar manner as presented by Hashimoto and colleagues. Since the method measures the µHbO_2_sat at each RBC position in the vessel, OxyTools makes a measurement of the µHbO_2_sat at the entrance and at the exit of each vessel, making it possible to measure the amount of oxygen extracted as measured previously in an experimental setting using trans-illumination in the rat skeletal muscle.^[^^36^^]^

An independent correlation was demonstrated between µHbO_2_sat measured by fiber spectrophotometry and the mean µHbO_2_sat of dual-wavelength OxyCam-generated µHbO_2_sat images. Observation of the µHbO_2_sat dependent pseudo colors in the different generation micro vessels corresponded with the expected better µHbO_2_sat of upstream capillaries compared to the downstream venules, which colored blue.

In a commentary on the expected role of monitoring the microcirculation for the future of intensive care,^[^^8^^]^ Jacquet‑Lagrèze et al.^[^^16^^]^ discussed the drawback and pitfalls of HVM devices. These included the need for offline manual image analysis and the lack of validated automatic software. The availability of automatic software is an important need for being able to make a differential diagnosis as to the origin of the microcirculatory tissue perfusion alterations.^[^^10^^,^^11^^]^ The introduction of computer aided analysis software called AVA3.2 dedicated to HVM microcirculatory imaging made this possible however these required operator software interaction^[^^37^^]^ Subsequently attempts were made to develop automatic microcirculatory analysis software but these failed in several validation studies.^[^^38^^,^^39^^]^ The development of clinically validated MicroTools for automatic analysis of microcirculatory imaging software introduced by Hilty and co-workers solved this problem,^[^^20^^,^^24^^]^ although it was not mentioned in their commentary of Jacquet‑Lagrèze et al.^[^^16^^]^ Integration of MicroTools in OxyTools together with the novel online stabilization algorithm has resolved the mentioned drawbacks related to analysis of images. The authors further point out that a drawback of HVM devices is that it does not provide information regarding tissue oxygenation, such as NIRS does. The OxyCam, providing µHbO_2_sat images and validated in the current paper, offers the availability of perfusion and oxygenation information in each individual microvessel in images, which represents an important step forward in tissue perfusion monitoring. Such information about the heterogeneity of microcirculatory oxygenation cannot be extracted from single-point measurements of perfusion and oxygenation, such as plethysmography, Capillary Refill Time, and NIRS.

There are, however, several limitations of this study which need to be considered. The first and foremost limitation of the study was that it was carried out in a rat instead of human subject (preferably sublingually). It would have been difficult to impose hypoxic and hyperoxic steps in humans as we were able to do in a rat model to validate the Hb saturation measurements, which was the most important aim of the study. A further a limitation of the present study was the choice of the muscle as a measurement site. This location was chosen pragmatically because it is least prone to movement artifacts caused by mechanical ventilation. The choice of muscle as a measurement site is also a limitation due to the presence of myoglobin in the tissue cells. Its spectrum is very similar to that of Hb although its oxygen saturation characteristics are very different (p50 much lower in myoglobin than in Hb). Our µHbO₂sat imaging methodology takes this aspect into consideration by measurement of the optical density of the vessel RBC and right next to the vessel and applying correction method of Hashimoto of the background tissues allows a focused measurement of the µHbO_2_sat. The O2C measurement, however, cannot make such a distinction between the oxygen saturation of Hb and that of myoglobin, which can introduce an artifact if one is solely interested μHbO_2_sat. This distinction makes it plausible that the signal to noise ratio of the O2C measurements are much lower than that of single vessel Hb saturation measurement performed by the OxyCam. These limitations of comparing the O2C measurement to the OxyCam measurement shown in Figure 4B could account for the finding that comparison of the measurements do not follow the identity line.

## Conclusions

It is concluded that this study has validated the OxyCam to reliably measure microcirculatory perfusion and oxygenation using dual wavelength oximetry.

We envisage the future perspective of the clinical application of the OxyCam to be manyfold. Now the critical point at which a microcirculatory perfusion deficit, as observed in many conventional HVM studies in shock and sepsis, results in a reduction of μHbO_2_sat can be identified. Such a functional effect on oxygen transport in the microcirculation could indicate the need for resuscitation procedures to be initiated. The μHbO_2_sat measurement could then be used to confirm whether resuscitation procedures, such as fluid, vasopressor and blood transfusion indeed translate themselves to an improved microcirculatory oxygen delivery.^[^^8^^]^

We expect the quantitative evaluation of microcirculatory perfusion and oxygenation at the level of the tissues by the OxyCam to provide an important point-of-care bedside clinical tool for critically ill patients for diagnostic assessment of circulatory compromise as well as its therapeutic resolution.

## Acknowledgments

None.

## Funding

The development of the OxyCam was supported by an EU Eurostar grant awarded to 10.13039/501100014979Active Medical (grant number ESTAR21214).

## Ethics Statement

Not applicable

## Conflict of Interest

The authors declare the following financial interests/personal relationships which may be considered as potential competing interests: The OxyCam has been developed by Active Medical BV, Leiden, of which CI, HK, and MH are respectively CSO, CEO, and CTO. In this capacity, they hold shares in Active Medical BV. Given his role as Editorial Board Member, Can Ince had no involvement in the peer-review of this article and has no access to information regarding its peer-review. Full responsibility for the editorial process for this article was delegated to another journal editor. The other authors have no declared interest.

## Data Availability

The data sets generated during and/or analyzed during the current study are available from the corresponding author upon reasonable request.

## CRediT authorship contribution statement

**Can Ince:** Conceptualization, Supervision, Writing – original draft. **Bülent Ergin:** Methodology, Formal analysis. **Jonathan Montomoli:** Software. **Mario Döring:** Conceptualization, Software. **Noor Boegborn:** Formal analysis. **Wijnie van Dam:** Formal analysis. **Giovanny Marchena:** Software. **Olcay Dilken:** Formal analysis. **Arie de Vos:** Methodology. **Husain Khaki:** Funding acquisition. **Diederik Gommers:** Funding acquisition. **Matthias Peter Hilty:** Conceptualization, Software, Project administration.
